# Antimutagenic effects of a tea made from Actinidia arguta, sarunashi in Japanese, and its inhibitory effects on the formation of aberrant crypt foci induced by 1,2-dimethylhydrazine in mice

**DOI:** 10.1186/s41021-025-00348-6

**Published:** 2025-12-22

**Authors:** Yusuke Saiki, Naoko Miyake, Sakae Arimoto-Kobayashi

**Affiliations:** 1 Faculty of Pharmaceutical Sciences, Okayama University, Okayama, Japan; 2 Graduate School of Medicine, Dentistry and Pharmaceutical Sciences, Okayama University, Okayama, Japan

**Keywords:** Tea, *Actinidia arguta*, sarunashi, Anticarcinogenicity, Aberrant crypt foci, Ames test

## Abstract

We previously determined the antimutagenic activity of the juice of *Actinidia arguta* (hereafter referred to as sarunashi-juice) using the Ames test. The anticarcinogenic effect of sarunashi-juice was observed in lung and skin tumorigenesis in mice. We hypothesized that tea prepared from the leaves and twigs of *A. arguta* (hereafter referred to as sarunashi-tea) might also have antimutagenic and anticarcinogenic properties. We investigated the antimutagenic activity of sarunashi-tea using the Ames test, and its preventive effects on the formation of aberrant crypt foci (ACF) induced by 1,2-dimethylhydrazine (DMH) in mice. Antimutagenic results against aflatoxin B_1_, benzo(a)pyrene (B(a)P), 2-amino-3,8-dimethylimidazo[4,5-*f*]quinoxaline (MeIQx), 3-amino-1-methyl-5*H*-pyrido[4,3-*b*]indole (Trp-*P*-2) and 1-methyl-6-phenyl-1*H*-imidazo[4,5-*b*]pyridin-2-amine (PhIP) were observed with the administration of sarunashi-tea. The amounts of sarunashi-tea needed for 50% inhibition (ID_50_) toward B(a)P, MeIQx, and Trp-*P*-2 were 2.9, 2.5, and 6.3 times higher, respectively, than that of sarunashi-juice. The antimutagenic components present in the juice and tea prepared from sarunashi may be similar chemicals. However, the concentration of the antimutagenic substances may be 2.5–6.3 times lower in sarunashi-tea than in sarunashi-juice. The total number of ACF was significantly reduced in mice treated with DMH in the presence of sarunashi-tea compared to that in mice treated with DMH in the absence of sarunashi-tea. Further investigation of the mechanisms of antimutagenicity and anti-ACF formation and identification of active components in sarunashi-tea will be worthwhile.

## Background

Green tea, made from the leaves and twigs of *Camellia sinensis*, is known to have anticancer and anti-inflammatory effects [1, 2]. In traditional Japanese medicine, extracts or decoctions of plant leaves, bark, and other parts of plants, such as *Geranium thunbergii* (gennoshoko in Japanese) [3, 4] and *Swertia japonica* (senburi in Japanese) [3, 5], are used as remedy-tea. Zhang et al. [6] summarized the phytochemical and healthy properties of *Actinidia arguta* (sarunashi in Japanese), and reported that the antitumor effects were mainly observed as inhibiting the proliferation and growth of tumor cells, promoting tumor cell apoptosis, enhancing the body’s immunity, and alleviating symptoms. Previously, we demonstrated the antimutagenic activity of juice of *A. arguta* (hereafter referred to as sarunashi-juice) using the Ames test, and observed the anticarcinogenic effect of a topically applied partially purified fraction of sarunashi-juice on skin tumorigenesis in mice induced by treatment with 7,12-dimethylbenz(a)anthracene (DMBA) and 12-*O*-tetradecanoylphorbol-13-acetate [7]. We also found that oral administration of sarunashi-juice significantly reduced 4‑(methylnitrosamino)‑1‑(3‑pyridyl)‑1‑butanone–induced lung tumorigenesis in A/J mice [8].

Colorectal cancer is the second most common fatal malignancy in Japan [9]. Aberrant crypt foci (ACF) are thought to be the earliest identifiable neoplastic lesions in a colon carcinogenetic model, and these lesions are generally considered useful preneoplastic biomarkers in mice and rats exposed to carcinogens [10, 11]. Crude α-mangostin administered through diet significantly inhibited the induction of ACF in 1,2-dimethylhydrazine (DMH)-treated rats [12], and treatment with a COX-2 inhibitor significantly inhibited the appearance of ACF, implying chemopreventive potential [13]. We hypothesized that tea prepared from the leaves and twigs of *A. arguta*, hereafter referred to as sarunashi-tea, may also have antimutagenic and anticarcinogenic properties.

In the present study, we investigated the antimutagenic activity of sarunashi-tea against aflatoxin B_1_ (AFB1), benzo(a)pyrene (B(a)P), DMBA and heterocyclic amines (2-amino-3,8-dimethylimidazo[4,5-f]quinoxaline (MeIQx), 3-amino-1-methyl-5 H-pyrido[4,3-b]indole (Trp-*P*-2) and 1-methyl-6-phenyl-1 H-imidazo[4,5-b]pyridin-2-amine (PhIP)) using the Ames test, and the preventive effects of sarunashi-tea on the formation of ACF induced by DMH in mice.

## Methods

### Materials

Leaves and twigs of *A. arguta* were obtained from Shin-jo village in Okayama Prefecture (Japan) in June. Processing of leaves (85%) and twigs (15%) of *A. arguta* for tea was outsourced to the Okayama plant of Marubishi Co., Ltd (Niimi-city, Okayama, Japan). The obtained tea leaves (2 g) of *A. arguta* were placed in 200 mL of hot water and kept at 98 °C for 2 min. Tea solution was filtrated through a tea-filter, and the obtained tea (hereafter referred to as sarunashi-tea) was stored at −20 °C until use. AFB1, B(a)P, DMBA, MeIQx, Trp-*P*-2, PhIP, and the supernatant fraction of rat liver homogenate prepared using phenobarbital and 5,6-benzoflavone were purchased from FUJIFILM Wako Pure Chemical (Osaka, Japan). The DMH was purchased from Kanto Chemical Co., Inc. (Tokyo, Japan). *Salmonella enterica subspecies I*, serovar Typhimurium (*Salmonella typhimurium*) strain TA98 and strain TA100 were gifts from Dr. B. N. Ames of the University of California, Berkeley [14].

## Animals

Mice (Cr1J: CD1 (ICR) male, 4-weeks old) were purchased from The Jackson Laboratory Japan, Inc. (Yokohama, Japan). Five mice were housed in animal compartments per cage and randomly divided into treatment groups at least 1 week before the start of the experiment. Mice had free access to pellets of murine chow (MF powder, Oriental Yeast Co., Ltd., Tokyo, Japan) and water with optimal air exchange and a 12-h light/12-h dark cycle at a constant room temperature of 20 °C. All the experiments were conducted in accordance with the Guidelines for Animal Experiments of Okayama University Advanced Science Research Center (permission no. OKU- 2018031, 2021462, 2022280, 2022299 and 2022365) based on the Act on Welfare and Management of Animals (Act of Japan, No. 105 of October 1, 1973, and Amendment of Act No. 68 of 2005) and the Standards Relating to the Care, Management, and Alleviation of Pain and Distress of Laboratory Animals (Notice of the Ministry of the Environment No. 88 of 2006).

## Antimutagenicity test

Sarunashi-tea was sterilized by filtering through a 0.45 M Millex syringe filter (Millipore, Merck KGaA, Darmstadt, Germany). The effects of sarunashi-tea on AFB1, B(a)P, and DMBA-induced mutagenicity were investigated with *S. typhimurium* TA100 and those of Trp-*P*-2, MeIQx, and PhIP-induced mutagenicity were investigated with *S. typhimurium* TA98 both in the presence of the supernatant fraction of rat liver homogenates using the Ames test [14]. The preincubation method [15] was used in this study. The effects of the compounds on the mutagenicity were examined using a previously described procedure [7]. Briefly, the preincubation mixture (700 mL) was prepared by mixing the components in the following order: 100 mL of sarunashi-tea or water, 450 mL of the rat liver homogenate solution for metabolic activation, 100 mL of an overnight culture of bacteria, and 50 mL of a mutagen solution. After incubating for 20 min at 37 °C, molten agar was added, and the mixture was poured onto a minimal agar plate. The plates were incubated for 2 nights, and the resulting revertant colonies were counted manually. The individual mutagen doses used were determined as those resulting in > 400 revertant colonies per plate. When the number per plate exceeded 3000, the colonies in a certain square area were counted, and the total number of colonies on the plate was estimated from the average counts in five such areas. The mutagenic activity (percentage) was obtained as follows:

100 × [(revertants in the presence of sarunashi-tea) – (spontaneous revertants)]/[(revertants in the absence of sarunashi-tea) – (spontaneous revertants)].

All experiments were performed in triplicates and repeated twice. All plates were examined using a stereomicroscope to determine whether the background lawn was impaired.

## Detection of the formation of aberrant crypt foci (ACF)

Formation of ACF was detected as advised by Prof. Dr. Keiko Kataoka, Faculty of Medicine, Tokushima University, based on several previous studies [12, 16]. Briefly, 48 male CD1 mice were randomly divided into four experimental groups. Mice in groups 1 and 2 were administered subcutaneous injections of 0.1 mL of DMH (20 mg/kg body weight), and mice in groups 3 and 4 were administered subcutaneous injections of 0.1 mL of phosphate buffered saline (PBS) once a week for 2 weeks. One week after the second injection of DMH or PBS, mice in groups 2 and 3 were administered sarunashi-tea as a replacement for water until the end of the experiment. All the mice were fed a basal diet throughout the experiments. All the mice were carefully observed and weighed weekly during the experiment. The experiment was terminated at 20 weeks after the start of the experiment and all mice were euthanized by cervical dislocation. Colons were carefully collected, washed with saline, opened longitudinally, and then fixed with 10% buffered formalin. Colon tissues were stained with a 0.5% methylene blue solution and then placed on a glass plate. Using a light microscope at a magnification of 40 x, the ACF were counted according to the criteria described in the literatures [13, 17].

## Statistical analyses

Data are expressed as means ± standard deviation. Statistical analyses were conducted using Dunnett test in data interpretation (*p* < 0.05). Statistical analyses were performed using KaleidaGraph Version 4.5.3 (Synergy Software, Reading, PA, USA) and Excel statistics (SSRI Co. Ltd., Tokyo, Japan).

## Results

### Effects of sarunashi-tea based on the mutagenicity assayed using the Ames test

**Fig. 1:**
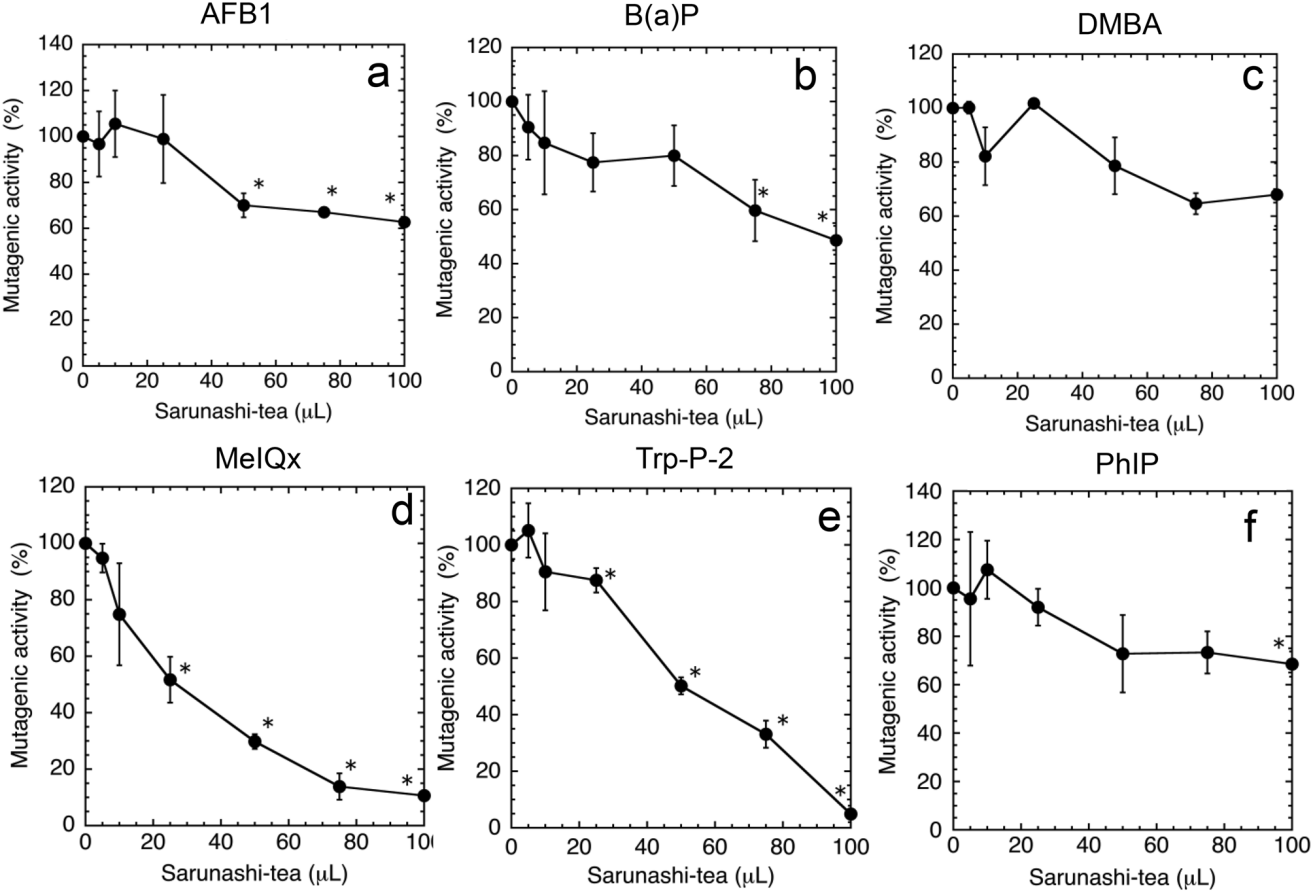
Effects of sarunashi-tea on the mutagenicity of aflatoxin B_1_ (AFB1) (**a**), benzo (**a**) pyrene (B(**a**)p) (**b**), 7,12-dimethylbenz (**a**) anthracene (DMBA) (**c**), 2-amino-3,8-dimethylimidazo[4,5-*f*]quinoxaline (MeIqx) (**d**), 3-amino-1-methyl-5*H*-pyrido[4,3-*b*]indole (Trp-*P*-2) (**e**), and 1-methyl-6-phenyl-1*H*-imidazo[4,5-*b*]pyridin-2-amine (PhIP) (**f**). Antimutagenicity was assayed with the Ames test using *S. typhimurium* TA100 and TA98. The experiment were performed in triplicates and repeated twice. Standard deviation (SD) is indicated with the bar (*n* = 3). *p < 0.05, significantly different from the mutagenic activity without sarunashi-tea

The antimutagenic properties of sarunashi-tea against mutagens were investigated using the Ames test. The mutagenicity of AFB1 and B (a)P, detected using TA100, and that of MeIQx, Trp-*P*-2, and PhIP, detected using TA98, were significantly reduced in the presence of sarunashi-tea (Fig. 1). The amount of sarunashi-tea needed for 50% inhibition (ID_50_) of the mutagenicity of B (a)P, MeIQx, and Trp-*P*-2 was approximately 100, 25, and 50 μL/plate, respectively (Fig. 1). The mutagenicity of AFB1 and PhIP did not decrease to 50% or less in the presence of sarunashi-tea (Fig. 1). The numbers of His^+^ revertants from *S. typhimurium* TA100 per plate found in the absence of the inhibitor was 1494 ± 23 for 0.5 nmole of AFB1, 1679 ± 199 for 10 nmole of B(a)P, and 521 ± 86 for 50 nmole of DMBA, and that from *S. typhimurium* TA98 was 1717 ± 96 for 50 pmole of Trp-*P*-2, 2336 ± 170 for 250 pmole of MeIQx, and 2853 ± 416 for 5.0 nmole of PhIP. The number of spontaneous revertants/plate (negative control) for *S. typhimurium* TA100 was 126 ± 13 and for TA98 was 26 ± 6. The numbers of His^+^ revertants per plate observed with 100 L of sarunashi-tea was 143 ± 35 with TA100 and 19 ± 8 with TA98, with no significant difference from those of spontaneous revertant frequencies. Therefore, sarunashi-tea did not show mutagenic activity against *S. typhimurium* TA100 or TA98. We examined all plates under a microscope and confirmed that the background lawn was not impaired, and no cytotoxicity was observed.

## Effects of sarunashi-tea on ACF formation

A total of 48 mice survived until the end of the experiment, and none of them developed colon tumors. The mean body weights of groups 1–4 at the end of the experiments were 56.5 ± 9.4, 55.1 ± 4.9, 52.2 ± 7.0, and 58.1 ± 5.1 g, respectively, and no significant differences were observed in body weight after treatment of DMH and/or sarunashi-tea. The number of ACF, ACF containing 1–3 crypts, and ACF containing more than four crypts per colon is listed in Table 1. The total number of ACF in group 2 was significantly reduced to 60.5% of that in Group 1. The number of ACF containing 1–3 crypts in Group 2 was also significantly decreased to 64.1% of that in Group 1. No ACF were observed in mice in groups 3 and 4.

**Table 1:** Effects of sarunashi-tea on the formation of aberrant crypt foci (ACF)

Group	Treatment	No. of animal examined	Total no. of ACF/colon	Total no. of ACF containing 1–3 crypts/colon	Total no. of ACF containing more than 4 crypts/colon
1	DMH + tap water	13	11.9 ± 5.6	9.2 ± 5.1	2.7 ± 2.7
2	DMH + Sarunashi-tea	15	7.2 ± 3.8 *	5.9 ± 3.0 *	1.3 ± 1.2
3	Sarunashi-tea alone	5	0	0	0
4	Tap water alone	15	0	0	0

*significantly different from group 1 by student’s t-test *p* < 0.05, respectively

## Discussion

The multistage induction theory is widely regarded as the mechanism of carcinogenesis. The functional components of edible plants are important for preventing the initiation of carcinogenesis. Previously, we found that sarunashi-juice inhibited the mutagenicity of AFB1, B(a)P, DMBA, MeIQx, Trp-*P*-2, and PhIP [7]. In the present study, similar antimutagenic results were observed for AFB1, B(a)P, MeIQx, Trp-*P*-2, and PhIP with sarunashi-tea. Similar antimutagenic components may be present in the juice and tea prepared from sarunashi. The amount of sarunashi-juice required to achieve an ID_50_ value with AFB1, B(a)P, DMBA, MeIQx, Trp-*P*-2, and PhIP was approximately 15, 35, 20, 10, 8.0, and 8.0 μL/plate, respectively [7]; ID_50_ values of sarunashi-tea toward B(a)P, MeIQx, and Trp-*P*-2 were 2.9, 2.5, and 6.3 times higher, respectively, than those of sarunashi-juice. The concentration of the antimutagenic substances may be lower in sarunashi-tea than in sarunashi-juice. Hiramatsu et al. [3] reported the antimutagenic activity of gennoshoko (*Geranium nepalense var. thunbergii*) and yomogi (*Artemisia vulgaris var. indica*) against Trp-*P*-2 and B(a)P. However, the extract of matatabi (*Actinidia polygama*) belonging to *Actinidia*, the same genus as sarunashi (*A. arguta*), did not show antimutagenicity against these mutagens. Antimutagenic components might not be distributed throughout the genus *Actinidia* but might be specific to *A. arguta* sarunashi.

Previously, we found that sarunashi-juice inhibited the induction of acute inflammation in mouse ears induced by 12-*O*-tetradecanoylphorbol-13-acetate, and suggested that some polyphenolic components in sarunashi-juice were the active components [7]. Non-steroidal anti-inflammatory drugs have been reported to reduce the risk of colorectal cancer [18]. Non-steroidal anti-inflammatory drugs are COX inhibitors and modulators of cell proliferation. Nabandith et al. [12] reported that dietary administration of a-mangostin (a major xanthone derivative in mangosteen pericarp (*Garcinia mangostana*)) inhibited the induction of ACF in rats treated with DMH, when compared to the only DMH-treated group, and reported that the inhibitory effects of a-mangostin may be associated with the inhibition of cell proliferation in the lesions. Resveratrol, a polyphenol found in grapes, inhibits the cell proliferation, triggers growth arrest, and induces apoptosis in a range of malignant cells [19]. Kineman et al. [20] reported that supplementation of resveratrol-glucoside accumulated in alfalfa (*Medicago sativa*) with a-galactosidase reduced the number of ACF in mice induced with azoxymethane, compare with those in mice provided a diet without alfalfa. They reported that polyphenolic compounds present in alfalfa plants protected against colon carcinogenesis. Our results suggested that the administration of sarunashi-tea inhibits the induction of ACF in mice treated with DMH, which might be related to the presence of polyphenolic components similar to those in sarunashi-juice. The major polyphenolic component of sarunashi-juice was identified as isoquercetin [21]. In our previous study, isoquercetin reduced 4‑(methylnitrosamino)‑1‑(3‑pyridyl)‑1‑butanone (NNK)-induced lung tumorigenesis, and phosphorylation of Akt, with or without epidermal growth factor stimulation, in A549 cells was significantly decreased following isoquercetin treatment [8]. Isoquercetin in sarunashi-tea might contribute the antimutagenic effects, and inhibitory effects on ACF formation induced by DMH in mice.

Further investigation of sarunashi-tea to determine the mechanisms of antimutagenicity and anti-ACF formation and identification of active components will be worthwhile.

## Conclusions

Antimutagenic effects were observed with sarunashi-tea, similar to those with sarunashi-juice, using the Ames test. Administration of sarunashi-tea also significantly reduced the total number of ACF in mice treated with DMH compared to that in mice treated with DMH in the absence of sarunashi-tea. Further investigation of sarunashi-tea to determine the mechanisms of antimutagenicity and anti-ACF formation and identification of active components will be worthwhile.

## Data Availability

No datasets were generated or analysed during the current study.

## Abbreviations

ACF: aberrant crypt foci
AFB1: aflatoxin B_1_
B(a)P: benzo(a)pyrene
DMBA: 7,12-dimethylbenz(a)anthracene
DMH: 1,2-dimethylhydrazine
ID_50_: the amount of substance needed for 50% inhibition
MeIQx: 2-amino-3,8-dimethylimidazo[4,5-*f*]quinoxaline
PBS: phosphate buffered saline
PhIP: 1-methyl-6-phenyl-1*H*-imidazo[4,5-*b*]pyridin-2-amine
Trp-*P*-2: 3-amino-1-methyl-5*H*-pyrido[4,3-*b*]indole
